# The effects of self-regulated learning strategies on academic procrastination and academic success among college EFL students in China

**DOI:** 10.3389/fpsyg.2025.1562980

**Published:** 2025-07-10

**Authors:** Xue Tao, Hafiz Hanif, Wang Lieqin

**Affiliations:** ^1^College of Foreign Languages, Baoji University of Arts and Sciences (BUAS), Baoji, China; ^2^Fakulti Pembangunan Manusia–Jabatan Pengajian Pendidikan, Universiti Pendidikan Sultan Idris, Tanjung Malim, Malaysia

**Keywords:** self-regulated learning, academic procrastination, academic success, EFL learners, structural equation modelling, bootstrapping, path coefficients, mediation effects

## Abstract

The failure of self-regulation lies at the core of academic procrastination, which poses a serious threat to academic success. This study examines the direct impact of self-regulated strategies on academic procrastination and academic success among university students. Additionally, it explores the mediating role of academic procrastination in the relationship between metacognitive strategies, time management, effort regulation, and students’ academic success. A fully quantitative study was conducted among 239 university students who learn English as a foreign language. In order to examine how self-regulated learning strategies can help university EFL students overcome academic procrastination and enhance academic success, 10 hypotheses were put to the test. The hypothesized model was tested using structural equation modeling (SEM) implemented in AMOS 23.0. Bootstrapping with bias-corrected confidence intervals was employed to evaluate the model’s path coefficients and mediation effects. The results reveal that there is a significant positive correlation between metacognitive strategies and academic success. Learning strategies such as effort regulation, metacognitive strategies, and time management are negatively associated with academic procrastination, and academic procrastination is negatively related to academic success. Regarding the mediation effects, it was found that effort regulation and time management have a significantly positive indirect influence on student’s academic success through the mediation of academic procrastination. Meanwhile, academic procrastination does not mediate the relationship between metacognitive strategies and students’ academic success. This study offers significant empirical evidence underscoring the critical role of self-regulated learning in academic success, while emphasizing the necessity of implementing targeted interventions to mitigate academic procrastination as a key impediment to student success. The implications of this study can assist educators or teachers in guiding students to appropriately apply self-regulated learning strategies and models to English learning.

## 1 Introduction

### 1.1 Background and significance of the study

In today’s globalized era, advanced English proficiency is critical for both academic success and career prospects (Fleckenstein et al., 2020; Fleckenstein et al., 2016). For EFL learners, this challenge is compounded by the exam-oriented nature of instruction (Cai, 2018), where language use remains largely classroom-bound, creating unique self-regulatory demands. English as a Foreign Language (DeFlorio et al., 2018) learner, particular from China, Brazil, and Nepal (Karbalaei, 2010; Yang and Farley, 2019), exhibit diverse goals and motivations that shape their commitment to language acquisition (Gardner and Lambert, 1959; Qin, 2007; Yu et al., 2018). The EFL context magnifies the well-documented relationship between academic success and self-regulated learning (SRL) (Cho et al., 2020). Language learners deploy diverse strategies (Cho et al., 2020; Oxford and Crookall, 1989), yet EFL instructors often prioritize knowledge transmission over strategic competence—a critical gap given that learning strategies training could facilitate the learning process to enhance learning outcomes and students’ English proficiency.

Academic procrastination, which was defined as the self-regulatory failure to act despite anticipated negative consequences (Li et al., 2021), it indicates characteristics such as a lack of conscientiousness, a high degree of impulsiveness, and difficulties in controlling one’s thoughts. Academic success has been spotted across diverse domains especially prominent in academic settings. It’s believed that academic procrastination affects 70–90% of university students. Roughly 80% college students admit to engaging in this self-sabotaging behavior, with around 90% delaying their tasks for at least 1 h each day (Klassen et al., 2008; Rahimi et al., 2016). Research indicates that 70% of university students are moderate delayers, while and 14% fall into the category of severe procrastinators (Chehrzad et al., 2017). Extensive studies have delved into various aspects regarding academic procrastination in general educational contexts. However, when the focus shifts to the specific group of university EFL students, a notable research gap emerges. Despite the importance of understanding how procrastination evolves and impacts this particular student population who are engaged in learning a foreign language within the university setting, there is paucity of relevant investigations. It remains challenging to trace comprehensive research that focuses on the development of procrastination among university EFL students (Ziegler and Opdenakker, 2018). Thus, there is an urgent call for more in-depth exploration to bridge this gap in the academic literature.

Learning procrastination is a widespread issue that plagues college students learning English. However, there is a scarcity of research exploring the influence of learning strategies on the academic performance of university English learners, especially when it comes to metacognitive strategies (Roick and Ringeisen, 2018). Additionally, only a harmful of studies have pinpointed that time management, which is part of resource management strategies, along with other strategic elements of self-regulated learning, could potentially predict students’ academic capabilities (Albaili, 1997; Bembenutty, 2009; Burlison et al., 2009; Kitsantas et al., 2008; Lynch, 2006; Wolters et al., 2017). It proves that effort regulation negatively relates to academic procrastination (Rakes and Dunn, 2010; Ziegler and Opdenakker, 2018). Nevertheless, very few studies have delved into the connection between academic procrastination and effort regulation, along with its links with self-efficacy and metacognitive structure (Komarraju and Nadler, 2013; Strunk and Steele, 2011; Sungur, 2007; Ziegler and Opdenakker, 2018). Within the sphere of higher education, the majority of research efforts has centered on the associations among academic procrastination, self-regulated learning, and academic performance.

Academic procrastination severely threatens students’ academic success. It gives rise to a decline in students’ academic success (Wolters and Brady, 2020), which is manifested as inferior course grades, unsatisfactory academic results, and, in extreme cases, even leading to course abandonment. Research in this area has uncovered that, as stated in the literature concerning academic procrastination, academic success serves as a significant negative predictor of academic procrastination. That is to say, a slump in academic success tends to exacerbate academic procrastination (Kandemir, 2014). Nevertheless, comparatively few studies have probed into the connection between the two in the context of academic success. By contrast, academic performance constituted one of the key determinants of academic success (Farruggia et al., 2016), making academic success a straightforward means of gauging college students’ learning outcomes.

### 1.2 Research aims

Past research has predominantly delved into the relationships among academic procrastination, self-regulated learning, and academic success from a multitude of perspectives covering diverse fields, regions, settings, nations, and involving individuals at various educational levels. More precisely, investigations focused on connections such as that between academic procrastination, metacognitive strategies, resource-management strategies, academic success. The present study endeavors to elucidate the interrelationships among academic procrastination, self-regulated strategies, and academic success within the context of college EFL students.

The goal of this present research was to formulate a framework for exploring the association between academic successes and self-regulated learning strategies, with academic procrastination acting as the mediating factor among university EFL students. In relation to the model that investigated the influence of motivational strategies on student performance via the mediation of academic procrastination, this particular study zeroed in on comprehending how resource-management strategies—which encompass effort regulation and time management—along with metacognitive strategies are connected to EFL students’ academic procrastination and their learning achievements.

This study concentrates on the following aims:

1 To explore the learning strategies like time management, effort regulation, metacognitive regulation that have correlations with both academic procrastination and academic success among college students who are learning English as a foreign language.
2 To create a framework to illustrate the relationship of metacognitive strategies, time management, and effort regulation on academic success, as well as the relationship of academic procrastination on metacognitive strategies, time management, effort regulation, and academic success among university EFL students.
3 To assess the direct impacts as well as the mediating effects of academic procrastination, metacognitive strategies, effort regulation, time management, and academic success among university EFL students.

### 1.3 Research questions and hypothesis

Over the past 30 years, self-regulated learning has held a prominent position in the realms of psychology and education. A multitude of models have been devised to delineate its ideal progression. It represents a sophisticated process integrating motivational, behavioral, and metacognitive elements. Ample research has verified that metacognitive self-regulation strategies exert a favorable impact on academic success. Specifically, prior investigations have ascertained that metacognitive self-regulation notably forecasts academic success, and academic success is closely tied to metacognitive strategies, efficient study time, internal resource-management strategies, and deep processing strategies being related to it.

Procrastination has been deemed a breakdown in self-regulation, giving rise to an adverse association between self-regulated learning and academic procrastination failure negatively linked to academic success (Loeffler et al., 2019a). One study indicated that academic procrastination thwarts academic success as it detrimentally influences both the volume and caliber of learning, culminating in subpar academic success. Grounded in earlier discoveries regarding self-regulation learning strategies concerning academic success and procrastination, the current study endeavors to probe the elements that shape the academic success of university EFL students. Furthermore, this research incorporates effort regulation, metacognition self-regulation, and time management in relation to academic procrastination and the success of EFL students at the university. The study also directs its focus on the ensuring research questions.

Procrastination is regarded as s self-regulation failure and negatively relates to self-regulated learning. Academic procrastination hinders academic success by negatively impacting learning quantity and quality. Based on previous findings about self-regulation learning strategies related to academic success and procrastination, the current study aims to explore factors influencing academic success of university EFL students, including aspects like effort regulation, metacognitive self-regulation and time management, and focus on specific research questions.

*RQ1:* For university EFL students, what are the relationships among metacognitive strategies, effort regulation, time management strategies, academic procrastination and academic success?

*RQ2:* For university EFL students, what is the framework of the direct relationships among academic procrastination, metacognitive strategies, effort regulation, time management, and academic success? What is the framework of the mediating role of academic procrastination in the effect of metacognitive strategies, time management, and effort regulation on academic success?

*RQ3:* For university EFL students, what are the direct effects among academic procrastination, metacognitive strategies, effort regulation, time management, and academic success? What are the indirect effects of metacognitive strategies, effort regulation, and time management on academic success by the mediation of academic procrastination?

This study aims to foster self-regulated learning strategies, which are expected to assist university EFL students in conquering their academic procrastination and attaining greater academic success. Subsequently, 10 hypotheses have been examined as follows.

Based on the study revealed that metacognitive strategies, internal resources, effort regulations, and time management were positively associated with academic success (Loeffler et al., 2019a). The following hypotheses were proposed:

Hypothesis 1: Metacognitive strategies are positively associated with academic success.

Hypothesis 2: Effort regulation is positively associated with academic success.

Hypothesis 3: Time management is positively associated with academic success.

The previous study showed that academic procrastination is negatively associated with effort regulation and metacognitive self-regulation (Ziegler and Opdenakker, 2018). Furthermore, a study revealed academic procrastination has a significantly negative relationship with time management (Loeffler et al., 2019b). The following hypotheses were presented:

Hypothesis 4: Metacognitive strategies bear a negative correlation with academic procrastination.

Hypothesis 5: Effort regulation has a negative association with academic procrastination.

Furthermore, previous study revealed that academic procrastination is significantly and negatively related with the time students utilize for studying effectively (Loeffler et al., 2019b). According to the research results, the following hypothesis is postulated:

Hypothesis 6: There is a negative relationship between time management and academic procrastination.

Previous studies held the view that academic procrastination correlates negatively with a student’s grade point average (GPA) (Grunschel et al., 2016). An investigation showed that motivational regulation strategies are positively related to students’ academic performance in an indirect manner, with academic procrastination acting as intervening variable. In conclusion, these studies indicate that a substantial positive connection between academic success and self-regulated learning, while showing that a markedly negative correlation with academic procrastination. Moreover, academic procrastination serves as a mediating factor in the relationship between academic procrastination and self-regulated learning. Grounded in previous discoveries, the subsequent hypotheses were put forward:

Hypothesis 7: Academic procrastination bears a negative relationship to academic success.

Hypothesis 8: Academic procrastination mediates the impact of metacognitive strategies on academic success.

Hypothesis 9: Academic procrastination mediates the effect of effort regulation on academic success.

Hypothesis 10: Academic procrastination mediates the effect of time management on academic success.

## 2 Literature review

Academic procrastination is a vital problem for it is intertwined with academic success, and there is no exception for university EFL students’ learning success. Since academic procrastination is not an independent phenomenon, it is complex and entangled with the main components of self-regulated learning strategies. This part aimed to learn how self-regulated learning strategies impact the academic success of students who engage in academic procrastination.

### 2.1 Strengths, weaknesses, and research gaps

This part effectively synthesizes a diverse range of studies from EFL contexts, highlighting the reciprocal relationship between self-regulated learning and academic procrastination. Citing research on the impact of goal-setting, time management, and metacognitive strategies on procrastination behaviors provides a solid foundation for understanding the key factors at play. The recognition of cultural adaptations in self-regulation, for instance, EFL students relying more on peer collaboration and teacher guidance, showcases the ability to consider context-specific elements, which is crucial for developing targeted interventions.

One significant weakness is the lack of in-depth exploration of the underlying mechanisms. While the studies identify associations between different factors, there is limited discussion on how exactly self-regulated learning strategies influence academic procrastination at a cognitive or psychological level. For example, although it is mentioned that language anxiety can lead to procrastination in EFL learners, the review does not delve into the specific cognitive processes through which anxiety affects self-regulatory efforts. Another weakness is the relatively narrow focus on a few types of self-regulated learning strategies. There is a greater emphasis on metacognitive and resource-management strategies, with less attention given to motivational strategies and their role in the context of EFL learning and procrastination.

A more specific research gap lies in the need to develop and test context-specific interventions that combine multiple self-regulation learning strategies. Given the unique challenges faced by EFL learners, such as language anxiety and cultural dissonance, there is a lack of research on how to design comprehensive programs that address these issues while enhancing self-regulatory capacities. Another gap is in exploring the long-term effects of self-regulated learning strategies on reducing procrastination and improving academic success. Most existing studies focus on short-term outcomes, and understanding the sustained impact of these strategies is crucial for educational practice.

Numerous studies in EFL contexts have confirmed the reciprocal relationship between self-regulated learning and academic procrastination. Previous studies showed that students with higher levels of goal-setting and time management, the key elements of self-regulated learning, were significantly less likely to procrastinate on English writing tasks. Zimmerman’s social-cognitive theory of self-regulation also demonstrated that metacognitive strategies, such as planning and monitoring, can mitigate procrastination behaviors. Other studies observed that EFL students relied more on peer collaboration and teacher guidance for self-regulation, unlike the individualistic approach often emphasized in Western research. This cultural adaptation highlights the need for context-specific interventions to address procrastination in foreign language learning (Yuxia et al., 2024).

Unique expressions of academic procrastination and self-regulation also emerge in EFL settings. Research revealed that EFL learners often procrastinate due to language anxiety, which is less prominent in native language learning. The pressure to produce error-free output, coupled with limited exposure to authentic language input, can impede self-efficacy and trigger procrastination (Kharrazi and Ghanizadeh, 2023). Additionally, institutional factors like large class sizes and exam-oriented teaching in many EFL classrooms, may undermine students’ autonomy in regulating their learning processes. These findings underscore the importance of integrating language-specific and context-sensitive strategies into self-regulated learning frameworks for EFL students.

Furthermore, challenges specific to EFL environments, such as cultural dissonance and linguistic barriers, significantly influence self-regulation and procrastination. Future research should explore how to reconcile cultural differences and design effective interventions that enhance EFL learners’ self-regulatory capacities while reducing procrastination tendencies.

### 2.2 Self-regulated learning

Self-regulated learning constitutes an active learning procedure that encompasses utilization of strategies and metacognition (Wang et al., 2019; Zimmerman, 1990). It has been drawing growing attention due to its significance in forecasting academic success. A similar definition believes self-regulated learning as an active process wherein learners systematically employ behavioral strategies, motivational strategies, and metacognitive strategies (Palos et al., 2019; Rotgans and Schmidt, 2009; Zimmerman, 1990). In recent years, the self-regulated learning concept has received escalating emphasis in both educational practice and research as it is acknowledged as a constituent of academic success (De Smul et al., 2018; Winne, 1997). Accordingly, students who can effectively use self-regulated strategies well during their learning stand a greater chance of attaining academic success.

To illustrate, in EFL learning, metacognitive strategies play a crucial role. When EFL students are tasked with writing an essay, planning strategies could involve outlining the main points and organizing the structure before starting to write. Monitoring strategies would be used to check if the writing is on topic, follows the correct grammar, and conveys the intended meaning. These metacognitive strategies are closely related to resource-management strategies. Time management, a part of resource-management, ensures that the student allocates enough time for each stage of the writing process, from planning to revising. Effort regulation, another aspect, enables students to stay focused and continue working on the essay even when facing difficulties, such as struggling to find the right words or encountering complex grammar structures.

Based on the social cognition theory, there exist 14 self-regulating learning strategies that can be categorized into four domains: metacognitive knowledge, resource management, motivational belief, and cognitive engagement (Anthonysamy et al., 2020). Metacognitive, one of the vital factors of self-regulated learning, including monitoring, regulating, and planning cognitive strategies use (Liu et al., 2020), which could let students regulate their behaviors in a specific learning context. Resource-management strategies are mainly composed the main sub-scales like time environment and effort regulation (Broadbent and Fuller-Tyszkiewicz, 2018).

Time management means arranging students’ time to improve the likelihood of realizing crucial targets within a given time. By managing the time successfully one can study highly effectively and productively. It showed that time management significantly predicted the performance of students (Zheng and Zhang, 2020). Procrastination, in contrast, reflects procrastinating behaviors and unnecessary delays, often seen as an abuse of time, that finally hinder the ability to achieve goals and limit individual performance (Wolters et al., 2017).

Effort regulation was considered a resource management strategy, signifying the capacity to exert effort even when a task is deemed extremely uninteresting, challenging, or wearisome (Pintrich, 1991; Ziegler and Opdenakker, 2018). Effort regulation has been regarded as an essential precondition for the implementation of metacognitive learning behaviors. Many literature has shown that effort regulation predicts and enhances the learning outcomes of students (León et al., 2015; Ruiz-Alfonso et al., 2020) and it had a stronger negative correlation with procrastination.

### 2.3 Academic success

Academic success raises from the learning process and belonged to the learning outcomes, which stands for students’ learning quality and quantity. In line with Astin’s I-E-O model, academic success is characterized as encompassing academic achievement, the accomplishment of learning goals, performance, and satisfaction. Academic achievement is the most commonly used measurement, usually measured in GPA and grades (by assignment or course grades). It can be considered that grades serve as an excellent quantitative overview of students’ success. Similarly, the academic success of students can be judged by their performance on standardized examinations. Other instruments for measuring academic success, the School Connectedness Scale (SCS) was employed to gauge students’ satisfaction with school, the Learning and Performance Self-efficacy scale under MSLQ motivation belief were used to assess students’ self-efficacy (Chew et al., 2020).

Academic success goes beyond just high grades in the EFL learning context. Students who can communicate effectively in English in real-life situations like participating in international conferences or having conversations with native speakers, have achieved a form of academic success related to practical language skills. The ability to understand and analyze English literature which demonstrates a deeper level of language proficiency and cultural understanding. These different manifestations of academic success are all influenced by elf-regulated learning strategies and are affected by academic procrastination.

### 2.4 Academic procrastination

Academic procrastination is when one delays completing an academic task or assignment usually because one does not want to do them. Essentially represents a failure in self-regulation since students unsuccessfully control their deeds or behavior in the study. This issue has now become a general problem among college students. It is considered that there is more than 70% of university students procrastinate on study tasks (Chen, 2019; O'Brien, 2002; Steel, 2007). Academic procrastination is regarded as trouble both to the self-regulation capabilities of college students and their pursuit of academic success (Hailikari et al., 2021; Zhang et al., 2018). There is a need for a comprehensive study of factors affect academic procrastination and the ways to reduce this phenomenon.

Academic procrastination can take on unique forms in EFL learning. Students may delay practicing English speaking because of the fear of making mistakes in pronunciation or grammar in front of others. This fear can stem from language anxiety, which is exacerbated by the pressure to perform well in a foreign language. What’s more, the complexity of English grammar rules or the difficulty of understanding idiomatic expressions can also lead to procrastination in tasks such as reading English novels or writing academic essays in English.

### 2.5 Relations between concepts

#### 2.5.1 Metacognitive self-regulation and academic procrastination

Metacognitive self-regulation forms a sub-component of the broader concept of self-regulation. By applying metacognitive strategies, regulating oneself successfully enables one to adjust his or her performance, monitor his or her actions, and control his or her behavior which is necessary to achieve a set goal. Metacognitive self-regulation is considered to be a crucial determinant of academic success (Ziegler and Opdenakker, 2018). In this connection, Baumeister and Tice consider procrastination as an indicator of weak self-regulation, and numerous studies corroborate the stance that lapses in self-regulation precipitate procrastination.

When EFL students lack metacognitive self-regulation in EFL learning, they may not plan their study effectively. For example, they might start reading an English text without setting clear goals about what they want to learn from it or without considering how to break it down into procrastination. On the other hand, students who use metacognitive strategies, such as self-questioning while reading to check their understanding, are more likely to stay on track and avoid procrastination.

#### 2.5.2 Resource-management strategies and academic procrastination

As an important resource management strategy, time management belongs to SRL and is considered unsuitable for unnecessary and anxiety-causing delays in completing academic work, which represents the traditional view of procrastination. Students who demonstrate more effective and increased time management abilities generally exhibit reduced degrees of conventional procrastination (Wolters et al., 2017). Fostering self-regulated learning habits to overcome academic procrastination showed that lower subjective procrastination ratings are caused by time management. Investigations unearthed that effort regulation has a stronger negative association with academic procrastination (Ziegler and Opdenakker, 2018).

Poor time management can lead to procrastination in the EFL context. If EFL students do not allocate enough time for daily English vocabulary practice, they may end up cramming before an exam, which is a form of procrastination. Effort regulation also plays a role. If students are not motivated enough to put in the effort to learn difficult English grammar structures, they may procrastinate on grammar exercises. However, if they can regulate their effort by rewarding themselves after completing a challenging grammar task, they are more likely to avoid procrastination.

#### 2.5.3 Academic procrastination and academic success

Academic procrastination has for a long time been recognized as a hindrance to academic success of students. Procrastination is regarded as a hindrance as it diminishes the quality and quantity of learning, while increasing students’ negative outcomes and the stress severity. Previous study has established a inverse relationship between academic success and the act of procrastinating in academics (Üztemur, 2020).

Academic procrastination can significantly impact academic success in EFL learning. Students procrastinates on writing English essays may not have enough time to revise and improve their work, resulting in lower grades. Moreover, procrastination can lead to increased stress, which can further impair language learning. On the other hand, students who avoid procrastination and regularly practice English speaking, writing, reading, and listening are more likely to achieve better academic results in EFL.

#### 2.5.4 Metacognitive self-regulation and academic success

Metacognitive strategy constitutes a vital element within the framework of self-regulated learning. It empowers learners to regulate, plan, and scrutinize their cognitive processes (Hayat et al., 2020; Pintrich and De Groot, 1990; Şen, 2016). At present, learners who use metacognitive learning strategies more proficiently are able to formulate superior learning plans, supervise and assess their comprehension and assimilation of study materials effectively, demonstrate greater responsibility, identify and address their issues, and strive to expand further (Hayat et al., 2020). Theory and research have affirmed the significant role that metacognitive learning strategies fulfill in achieving academic success. Many empirical studies have furnished evidence that learning strategies yield a positive influence on learning outcomes, thereby reaffirming the pivotal part of metacognitive learning strategies in academic success.

In EFL learning, metacognitive self-regulation is essential for academic success. An EFL student who uses metacognitive strategies to plan their English vocabulary learning, such as creating a weekly vocabulary list and setting goals for memorization, is more likely to make steady progress and achieve netter results in English language proficiency tests. By regularly monitoring their vocabulary acquisition and adjusting their learning methods if necessary, they can optimize their learning process and enhance their academic success.

#### 2.5.5 Resource-management strategies and academic success

Previous research discovered that factors such as effective learning time, metacognitive strategies, deep processing strategies, and internal resource-management strategies are favorably correlated with learning success (Loeffler et al., 2019a). Based on the component of the self-regulated learning model, time management is a resource-management strategy (Schmitz and Wiese, 2006). It was shown that Learning strategies like time management bear a positive association with academic success.

In EFL learning, resource-management strategies contribute to academic success. Time management helps EFL students balance different aspects of language learning, such as spending appropriate time on listening, speaking, reading, and writing exercises. Effort regulation ensures that students are willing to put in the necessary effort to learn English, even when it is challenging. An EFL students who manages their time well and regulates their effort to practice English speaking every day is more likely to improve their speaking skills, which is an important aspect of academic success in EFL.

This part has explored the complex relationship between self-regulated learning strategies, academic procrastination, and academic success in the context of EFL learning. By critically analyzing the cited literature, its strength, weaknesses, limitations, inconsistencies, and specific research gaps were identified. The main and sub-concepts were expanded and clarified, which providing examples from EFL learning and establishing logical connections between them. Overall, the review highlights the importance of considering context-specific factors, developing comprehensive interventions, and further researching the long-term effects of self-regulated learning strategies on reducing procrastination and enhancing academic success for EFL students.

## 3 Methods

### 3.1 Participates

This study was conducted in universities of Shaanxi province in China, and all participants were EFL undergraduate students who were native speakers. In this study, a sample comprising 279 volunteers students, who were learning English as a foreign language from four universities in Shaanxi Province were investigated. The age of these participants spanned from 18 to 23 years. The research aims is to probe into the way in which resource-management and metacognitive strategies are associated with the academic success of university EFL students, with academic procrastination serving as the mediating factor. In this manner, simple random sampling, which belongs to the probability sampling methods, is the most suitable strategy for this study.

### 3.2 Instruments

Questionnaires with 5-point and 7-point Likert Scale and students’ course grades were used to collect data. Of all the 44 items, 17 had been removed because they did not fit well into the corresponding sub-scales.

#### 3.2.1 Academic procrastination

Tuckman Procrastination Scale-German (Grunschel et al., 2016; Stöber and Joormann, 2001; Tuckman, 1991), which contains 16 items adapted to the academic context, was translated into English for Chinese EFL students to assess academic procrastination. All items deal with delays in student learning-related tasks (Klingsieck, 2013; Grunschel et al., 2016). Responses are evaluated and assigned scores on a scale ranging from 1, which represents “strongly disagree,” to 5, indicating “strongly agree” (Grunschel et al., 2016).

#### 3.2.2 Self-regulated learning strategies

The Motivated Strategies for Learning Questionnaire represents a reliable and valid tool that is utilized to gauge the self-regulated learning strategies and motivational orientations of university students within a specific course (Hayat et al., 2020). The MSLQ (Pintrich, 1991; Shelton et al., 2019) is a self-report scale consisting of 81 items. Among these, there are 31 items are included in the motivation section, 31 items are in the learning strategies part that concerning students using various metacognitive and cognitive strategies, and 19 items of learning strategy section concerning student managing different resources (Pintrich, 1991). Students were requested to rate each item concerning their experience in a particular classroom. All the items were rated on a 7-point Likert scale, with the range from “1 = not at all true of me” to “7 = very true of me” (Shelton et al., 2019).

In this study, sub-scales like measured metacognitive strategies and the management of internal resources (including study time, effort regulation) were selected from MSLQ to measure these four sub-strategies.

##### 3.2.2.1 Metacognitive self-regulation

The sub-scale focused on cognitive and metacognitive strategies, which is sourced from the MSLQ and pertains to metacognitive self-regulation, encompasses 12 items. The responses for these items are graded on a 7-point Likert scale with endpoints of 1 (“not at all true of me”) and 7 (“very true of me”). The self-regulation and control aspects of meta-cognition belonging to MSLQ were focused on.

##### 3.2.2.2 Time and study environment

The sub-scale related to resource-management strategies, which is drawn from the MSLQ and concerns study time and environment, was employed. The scoring of this sub-scale relies on six items, each rated on a 7-point Likert scale with endpoints of 1 (“not at all true of me”) and 7 (“very true of me”). Time management includes planning, managing, and scheduling study time, so there are six items related to time management adopted in this study.

##### 3.2.2.3 Effort regulation

Effort regulation means the capacity to control the workflow associated with the school. Low scores show, for instance, that the students often stop working (for example, due to boredom) even though the task has not been finished.

The sub-scale for resource-management strategies that deals with effort regulation is founded on four items taken from the MSLQ and rated on a 7-point Likert scale. The scale ranges from 1 (“not at all true of me”) to 7 (“very true of me”) (Pintrich, 1991; Ziegler and Opdenakker, 2018).

#### 3.2.3 Academic success

To measure the outcome variable of academic success, a combination of English course grades, academic self-efficacy, and school satisfaction was utilized.

##### 3.2.3.1 Course grades

In the model of academic success, academic achievement was presented as a learning outcome that measures a student’s academic work quality, such as assignment grades, course grades, or GPA, because academic achievement described a student’s academic performance (Astin, 1990; York et al., 2015). A variety of academic performance indicators including assignment grades, examination grades, and self-reported GPA are used to measure academic success (Kim and Seo, 2015). Based on a comprehensive study of the best ways to examine academic success, GPA and grades have generally been used to measure academic success (Zeynali et al., 2019). The complicated basic I-E-O model also indicates that students’ learning in a specific course is supposed to be reflected in that grade (York et al., 2015). So the grades that students obtained in their courses were employed to test their academic success in English courses.

In this study, students’ final exam grades for English courses were one of the assessments used to measure academic success. The grades were recorded, so a high score indicates good academic success (Grunschel et al., 2016). In China, most universities adopt the hundred-mark system in the final examination. For the convenience of input and analyze statistics, the English examination grades were recoded into grades range from 1 to 5 [1 = excellent (90 ≤ grades≤100), 2 = good (80 ≤ grades<90), 3 = satisfactory (70 ≤ grades<80), 4 = sufficient (60 ≤ grades<70), 5 = inadequate/failed (grades<60)].

##### 3.2.3.2 Academic self-efficacy

The subscale of self-efficacy for learning and performance, failing within the Motivational beliefs of the MSLQ, was employed to gauge the self-efficacy of school students (Onoda, 2012; Yip, 2019; York et al., 2015). This subscale is composed of 8 items, and along them, 2 items from the 7-point Likert scale (ranging from 1 = “not at all true of me” to 7 = “very true of me”) that were relevant to this study.

##### 3.2.3.3 School satisfaction

The School Connectedness Scale (SCS) was used to measure student satisfaction. Three statements related to this research were selected from the School Connectedness Scale (SCS): “I am happy to be at this school,” “The teachers at this school treat students fairly,” and “I feel safe in my school.” For each statement, the response opinions were scored on a 5-point Likert scale, where 5 stood for “strongly agree” and 1 denoted “strongly disagree.”

### 3.3 Procedure

Structural Equation Modeling has become a preferred technique by interdisciplinary researchers and is increasingly a “must” for social science researchers.

**Figure 1:**
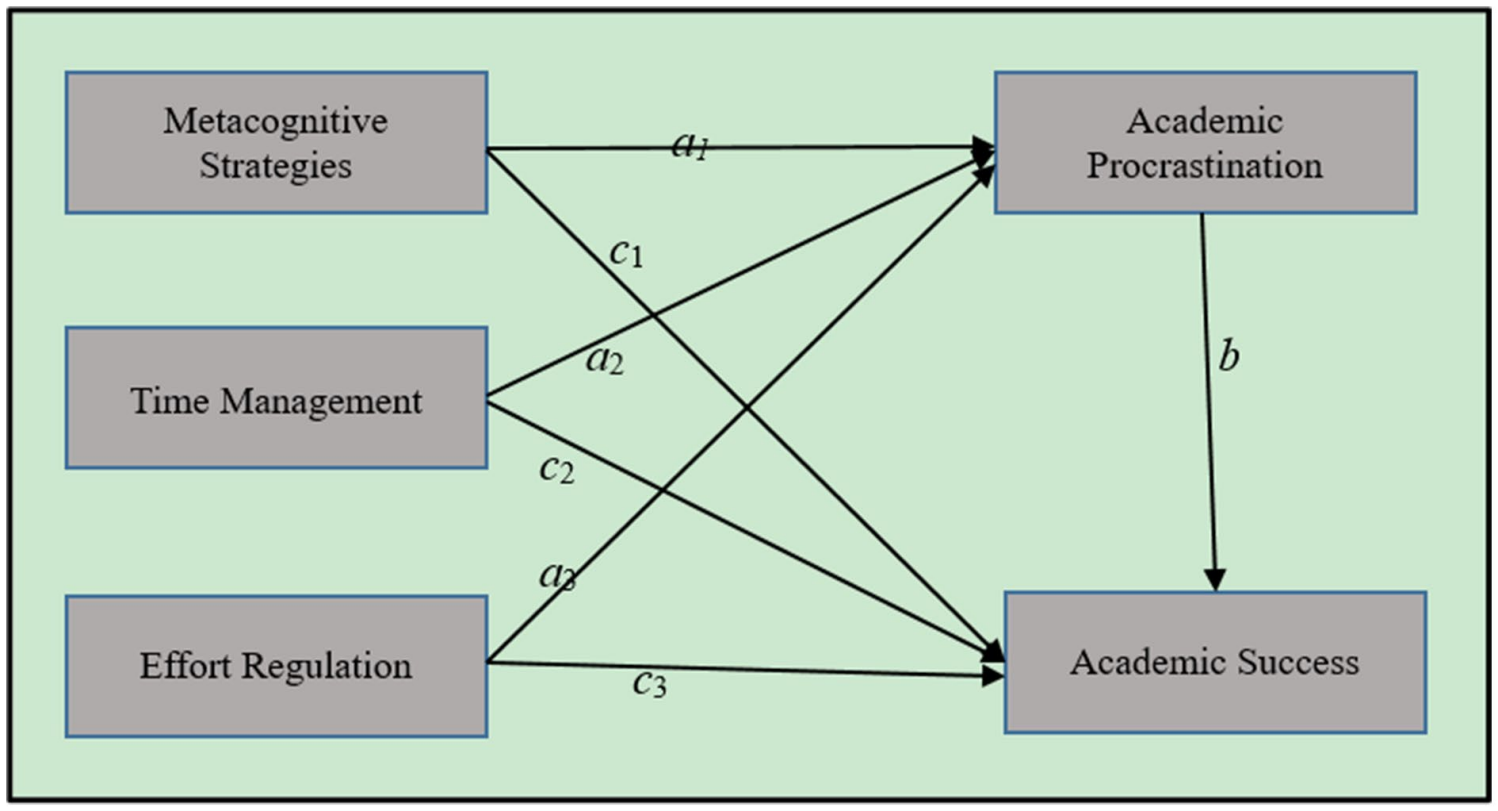
The assumed relation of the use of metacognitive strategies, effort regulation, and time management on students’ academic procrastination and academic success.

To examine the paths among the metacognitive strategies, effort regulation, time management, academic procrastination, and academic success, correlational findings were considered. Analyses based on the SEM model were carried showing the hypothetical relationship between the variables under study. The model depicted in Figure 1 illustrated the relationship between metacognitive strategies, effort regulation, time management, academic procrastination, and academic success among university EFL students.

The following figure described the effects of metacognitive strategies, effort regulation, and time management on academic procrastination and academic success, as well as the effect of academic procrastination on academic success (Grunschel et al., 2016; MacKinnon, 2008; Preacher and Hayes, 2008; Corkin et al., 2011): (1) Metacognitive strategies are significantly associated with academic procrastination (path *ɑ_1_*); Time management is significantly associated with academic procrastination (path *ɑ_2_*); Effort regulation is significantly associated with academic procrastination (path *ɑ_3_*); (2) Academic procrastination is significantly related to academic success (path *b*); (3) Metacognitive strategies are significantly associated with academic success (path *c_1_*); Time management is significantly associated with academic success (path *c_2_*); Effort regulation is significantly associated with academic success (path *c_3_*).

### 3.4 Data analyses

Five variables which are metacognitive strategies, effort regulation, time management, academic success, and academic procrastination were set in this study. For the path analysis, percentile bootstrapping and bias-corrected percentile bootstrapping were carried out at a 95% confidence interval with 2,000 bootstrap samples. To explore the direct and indirect effects of metacognitive self-regulation, academic procrastination, effort regulation, time management, and academic success, structural equation modeling was carried out in AMOS was utilized to test the 10 hypotheses.

**Figure 2:**
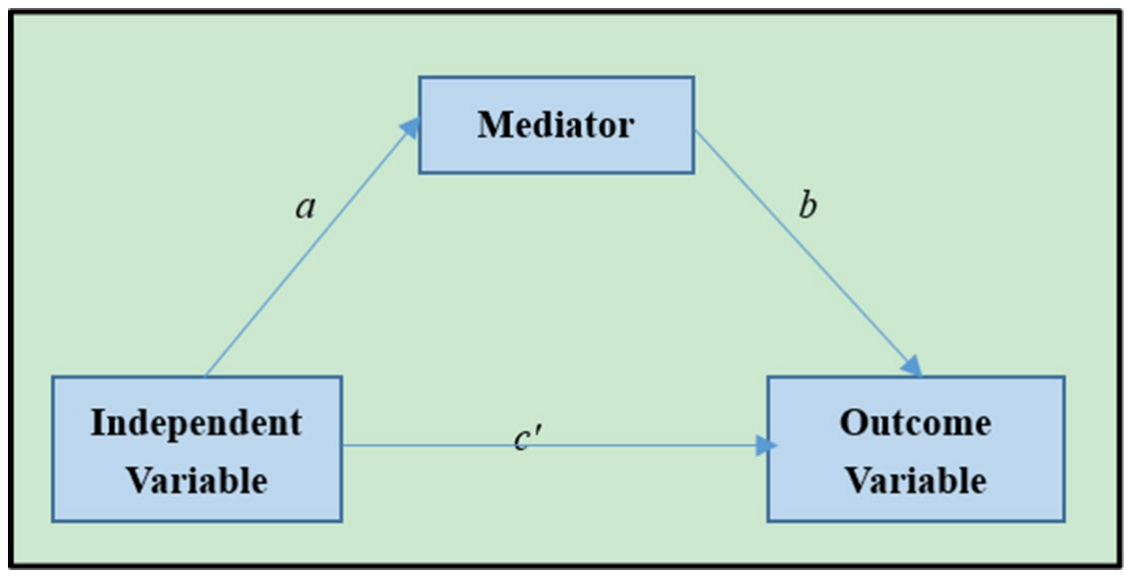
A simple mediation model.

Figure 2 presents a simple mediation variable model. In this model, a represents the coefficient of the independent variable in the mediation between the independent variable and the model prediction, *b* is the coefficient of the independent variable in relation to the model prediction outcome variable, and *c’* is also the coefficient of the independent variable and the model prediction outcome variable. In terms of path analysis, *c’* measures the direct effects of independent variables. In contrast, the product of a and b gauges the indirect effects of the independent variable on the outcome variable through mediation. If these three variables are observable, the total effect c = *c’ + ab* (Baron and Kenny, 1986; Hayes, 2009).

#### 3.4.1 Casual step

Bootstrapping aroused as the supplementary method to the causal steps approach, which has the best Type I error control and the highest power. It is one of the more robust and valid techniques for testing the effect of the intervention variable (Mackinnon et al., 2004; Williams and Mackinnon, 2008). Some SEM software (such as AMOS), Bootstrapping has achieved guided indirect effects. Bootstrapping repeats *k* times, where *k* is a fairly large number (usually at least 1,000), and then uses a *k* estimate to generate a *ci%* confidence interval. This process generates a bootstrap confidence interval based on percentage. The endpoints can be adjusted to produce a *bias-corrected* confidence interval. Whichever one you use, if zero does not fall between the upper and lower bounds, the analyst can assert that the indirect effect is not zero in the *ci%* confidence interval. This is tantamount to rejecting the null hypothesis which posits that the actual indirect effect is zero at the significance level of 100-*ci%*. Bootstrapping is being used more and more frequently (Hayes, 2009).

The present research conducted a fully quantitative study. In all the mediating analyses, academic procrastination was the mediating variable, metacognitive strategies, effort regulation, and time management were exogenous variables, and the academic success of EFL students was the outcome variable. CFA was applied to determine the measurement model, followed by SEM to fit the data and obtain the path coefficients of the hypothetical model. IBM SPSS Statistics 23.0 was utilized for data entry. SEM and AMOS 23.0 software were employed to test the hypothesis model. During the analysis, Bootstrapping and bias-corrected confidence intervals were utilized to test the hypothesis model.

## 4 Results

In covariance analysis and mean structure analysis, two crucial assumptions related to Structural Equation Modelling require continuous scale and multivariate normal distribution of data (Byrne, 2013). After reviewing empirical studies of non-normality in Structure Equation Modelling, West concluded four important findings (Byrne, 2013; West et al., 1995). First, as the data become more and more abnormal, the value of χ^2^ estimated by ML became huge. Second, consider the situation where the sample size is extremely small (even under multivariate normality conditions), both the ML and generalized least squares (GLS) estimators produce χ^2^ values slightly exaggerated values. In addition, non-normality became more pronounced as the sample size decreased (Anderson and Gerbing, 1984; Boomsma, 1982; Byrne, 2013). Third, fitting indices like the Tucker-Lewis Index (TLI) (Byrne, 2013; Tucker and Lewis, 1973) and Comparative Fitting Index (CFI) (Byrne, 2013; Bentler, 1990) are modestly underestimated when the data are abnormal (Byrne, 2013; Marsh et al., 1988). Finally, non-normality results in a low standard error, with the degree of underestimation varying from moderate to severe (Byrne, 2013). One way to deal with multivariate non-normal data is to employ a process called “the bootstrap” (Byrne, 2013; Yung and Bentler, 1996; Zhu, 1997).

### 4.1 Path analysis

The confirmatory analysis indicated that the hypothesized model was well-suited to the data. The percentile bootstrapping and bias-corrected percentile bootstrapping were conducted at a 95% confidence interval with 2,000 bootstrap samples to explore the direct effects of the dependent variables. To explore the direct relationships among metacognitive self-regulation, academic procrastination, effort regulation, time management, and academic success, Structural Equation Modeling using AMOS 23 was employed to test hypotheses 1 to 7.

#### 4.1.1 Direct effects of metacognitive self-regulation on academic procrastination and academic success

**Figure 3:**
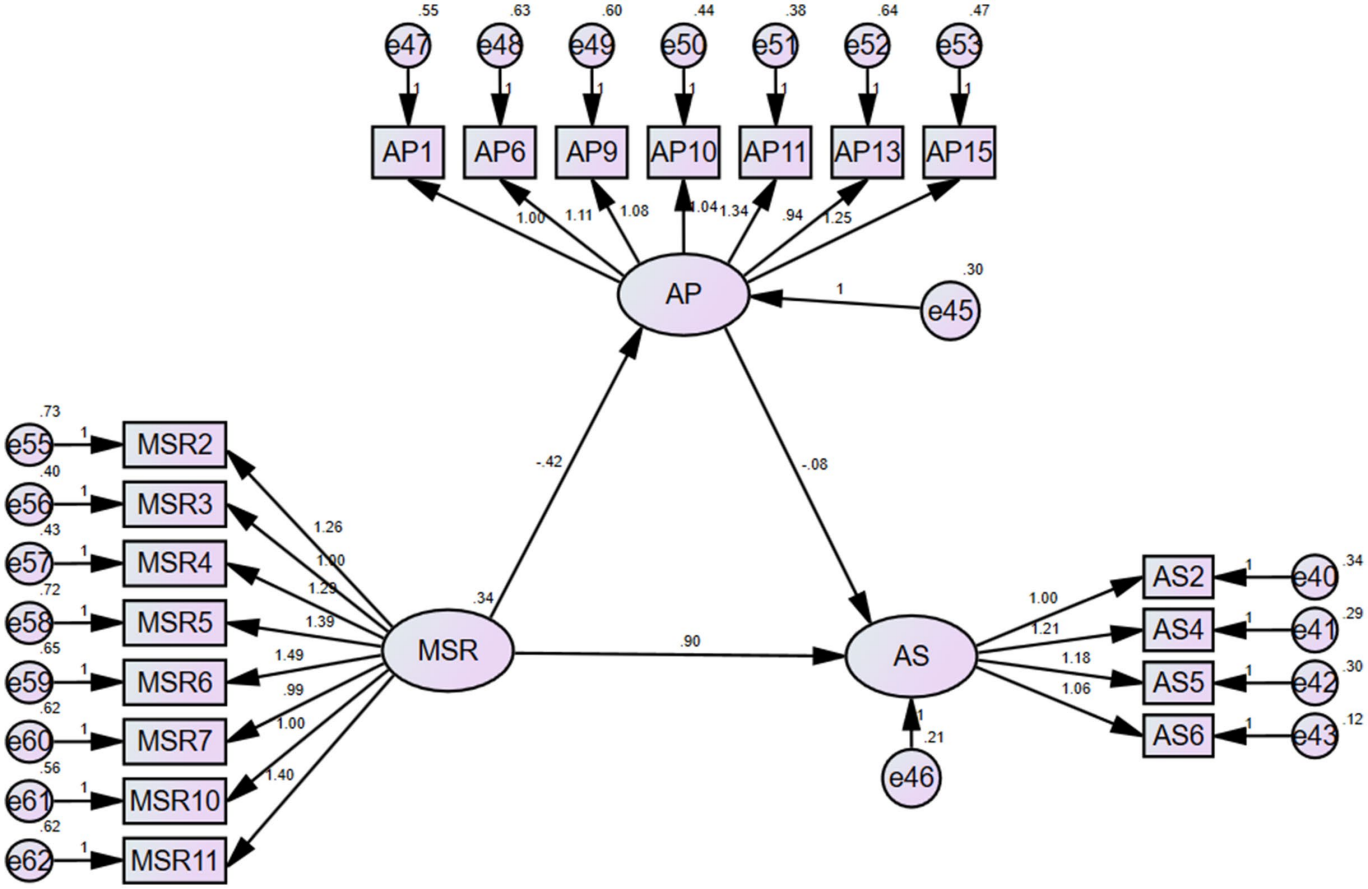
SEM model for MSR to AS.

Figure 3 and Table 1 show that metacognitive self-regulation exhibits a negative correlation with academic procrastination (*p*-value < 0.01, which was significant; the unstandardized estimate was −0.42; the standardized estimate was −0.406). Thus, Hypothesis 4 was confirmed. Metacognitive self-regulation also exerts a powerful and positive influence on academic success (*p*-value < 0.01, which was significant; the unstandardized estimate was 0.896; the standardized estimate was 0.731). Thus, Hypothesis 1 was confirmed.

**Table 1:** Path analysis for MSR.

Path	Unstandardized estimates	Standardized estimates	S.E.	C.R.	*p*
MSR → AP	−0.42	−0.406	0.086	−4.859	***
MSR → AS	0.896	0.731	0.11	8.164	***

***Represents that *p*-value < 0.01.

#### 4.1.2 Direct effects of effort regulation on academic procrastination and academic success

**Figure 4:**
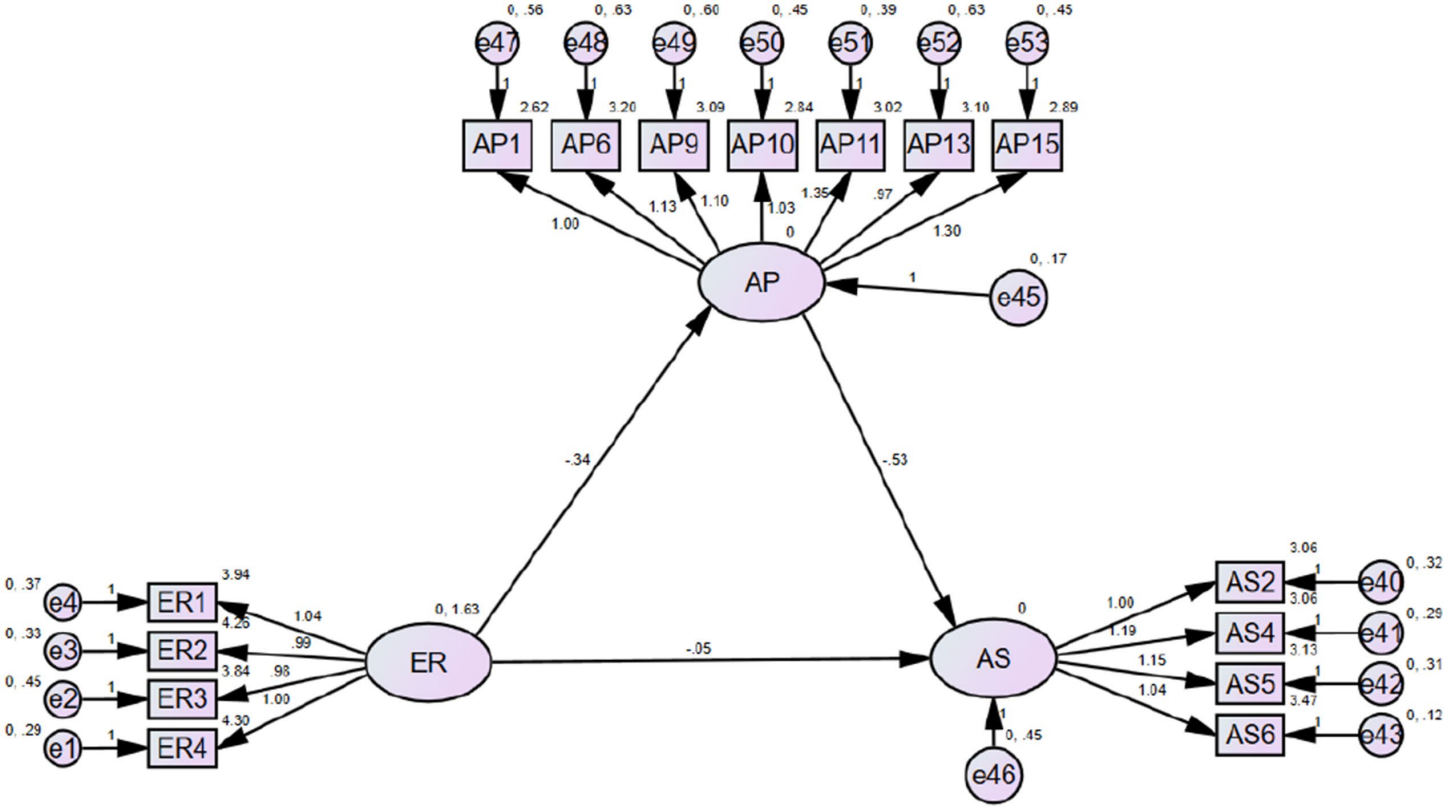
SEM model for ER to AS.

Figure 4 and Table 2 show that effort regulation is negatively correlated with academic procrastination (*p*-value < 0.01, which was significant; the unstandardized estimate was −0.336; the standardized estimate was −0.72). Thus, Hypothesis 5 was confirmed. However, the anticipated increase in academic success due to effort regulation was not realized (*p*-value > 0.01, which was insignificant). Thus, Hypothesis 2 was not confirmed.

**Table 2:** Path analysis for ER.

Path	Unstandardized estimates	Standardized estimates	S.E.	C.R.	*p*
ER → AP	−0.336	−0.72	0.04	−8.484	***
ER → AS	−0.054	−0.094	0.062	−0.867	0.386

***Represents that *p*-value < 0.01.

#### 4.1.3 Direct effects of time management on academic procrastination and academic success, academic procrastination on academic success

**Figure 5:**
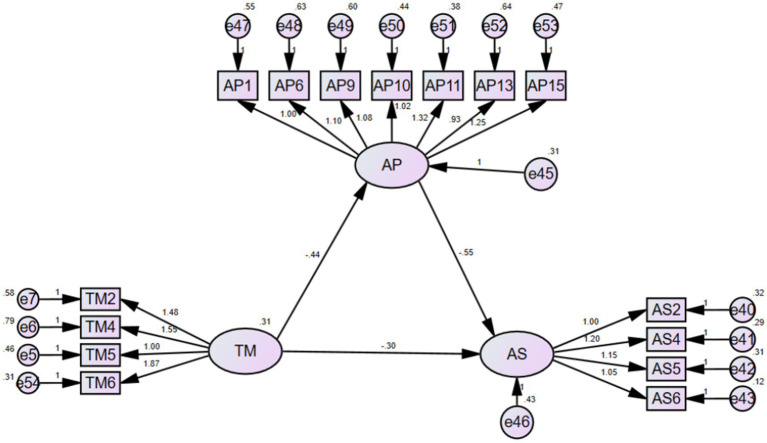
SEM model for TM to AS.

Figure 5 and Table 3 imply that time management is negatively correlated with academic procrastination (*p*-value < 0.01, which was significant; the unstandardized estimate was −0.44; the standardized estimate was −0.404). Thus, Hypothesis 6 was confirmed. Academic procrastination affects academic success negatively (*p*-value < 0.01, which was significant; the unstandardized estimate was −0.546; the standardized estimate was −0.458). Thus, Hypothesis 7 was confirmed. Nevertheless, the expected enhancement in academic success due to time management did not occur (*p*-value < 0.01, which was significant). Thus, Hypothesis 3 was not confirmed.

**Table 3:** Path analysis for TM.

Path	Unstandardized estimates	Standardized estimates	S.E.	C.R.	*p*
TM → AP	−0.44	−0.404	0.096	−4.569	***
AP → AS	−0.546	−0.458	0.109	−5.003	***
TM → AS	−0.302	−0.232	0.108	−2.783	0.005

***Represents that *p*-value < 0.01.

### 4.2 The mediating effects of academic procrastination

To study the indirect effects of dependent variables through mediations, percentile bootstrapping and bias-corrected percentile bootstrapping of 95% confidence interval with 1,000 bootstrap samples were performed (Taylor et al., 2008; Wang et al., 2014). Based on Preacher’s and Hayes’ suggestions, the confidence interval of the upper and lower bounds was computed to ascertain whether the indirect effects were significant (Preacher and Hayes, 2008; Wang et al., 2014). The Z-value exceeds 1.96, indicating a significant positive impact, and Z-value is lower than −1.96 showing significant negative effects (Lee, 2016).

As demonstrated in Table 4, the bootstrap test outcomes confirm that there are total effects and direct effects between metacognitive strategies, academic success, and academic procrastination. However, there was no mediating effect of academic procrastination between metacognitive strategies and academic success did not exist. The Z-value of total effects is 6.79, which is greater than 1.96, which means the total effects are significant. The Z-value of direct effects is 6.69, which is greater than 1.96, which means the direct effects are significant. The Z-value of indirect effects is 0.94, which is less than 1.96, which means there are no indirect effects, so academic procrastination does not significantly mediate the effect of metacognitive strategies on academic success (Hypothesis 8 was not confirmed).

**Table 4:** Unstandardized direct, indirect, and total effects of the hypothesized model.

Variable	Estimate			Bootstrapping			
		Product of coefficients		Bias-corrected 95% CI		Percentile 95% CI	
		SE	*Z*	Lower	Upper	Lower	Upper
			*Total effects*				
MSR → AS	0.930	0.137	6.79	0.698	1.243	0.706	1.258
			*Indirect effects*				
MSR → AS	0.034	0.036	0.94	−0.045	0.100	−0.041	0.101
			*Direct effects*				
MSR → AS	0.896	0.134	6.69	0.676	1.204	0.680	1.214

Unstandardized estimating of 1,000 bootstrap samples.

As shown in Table 5, the bootstrap test results confirm that there is a total effect and an indirect effect between effort regulation, academic procrastination, and academic success, but the direct effect did not exist. The Z-value of the total effect was 2.82, greater than 1.96, indicating that the total effect was significant. The Z-value of direct effects is −0.59, which is greater than −1.96, which means the direct effects do not exist. As depicted in Table 5, the Z-value of indirect effects is 2.25, which is greater than 1.96, indicating that the indirect effects exist. Therefore, academic procrastination notably mediates the impact of effort regulation on academic success (Hypothesis 9 was thus supported).

**Table 5:** Standardized direct, indirect, and total effects of the hypothesized model 1.

Variable	Point estimate			Bootstrapping			
		Product of coefficients		Bias-corrected 95% CI		Percentile 95% CI	
		SE	Z	Lower	Upper	Lower	Upper
			*Total effects*				
ER → AS	0.124	0.044	2.82	0.037	0.209	0.040	0.213
			*Indirect effects*				
ER → AS	0.178	0.079	2.25	0.044	0.344	0.052	0.352
			*Direct effects*				
ER → AS	−0.054	0.092	−0.59	−0.237	0.111	−0.255	0.103

Unstandardized estimating of 1,000 bootstrap samples.

As presented in Table 6, the bootstrap test results verified the presence of direct effects and mediation effects on academic procrastination, time management, and academic success. Still, the total effects of academic procrastination between time management and academic success did not exist. The Z-value of the total effect is −0.53, greater than −1.96, which means the total effect is nonexistent. The Z-value of the direct effects is −2.29, which is less than −1.96, which means the direct effects are significant. The Z-value of indirect effects is 3.33, which is greater than 1.96, which means the indirect effects exist. Thus, academic procrastination significantly plays a remarkably mediating role in the effect of time management on academic success (Hypothesis 10 was thus supported).

**Table 6:** Standardized direct, indirect, and total effects of the hypothesized model 2.

Variable	Point estimate			Bootstrapping			
		Product of coefficients		Bias-corrected 95% CI		Percentile 95% CI	
		SE	Z	Lower	Upper	Lower	Upper
			*Total effects*				
TM → AS	−0.062	0.117	−0.53	−0.308	0.170	−0.311	0.169
			*Indirect effects*				
TM → AS	0.240	0.072	3.33	0.121	0.411	0.118	0.397
			*Direct effects*				
TM → AS	−0.302	0.132	−2.29	−0.592	−0.070	−0.593	−0.071

Unstandardized estimating of 1,000 bootstrap samples.

## 5 Conclusion

### 5.1 Discussion

By analyzing the SEM of the direct effects of metacognitive strategies, effort regulation, and time management on academic success, the findings revealed a significant positive association between metacognitive strategies and academic success (Hypothesis 1 was confirmed). In contrast, no positive link was discovered between effort regulation and academic success (Hypothesis 2 was not confirmed). Additionally, there was no significant positive association between time management and academic success (Hypothesis 3 was not confirmed). This was in line with metacognitive strategies, internal resources, and effort regulations that have positive effects on academic success.

Hypothesis 2 and Hypothesis 3 were not supported, several potential reasons may account for these unexpected findings. One possible explanation for the lack of a positive relationship between effort regulation and academic success could be related to measurement issues. Effort regulation is a complex construct that involves an individual’s ability to sustain effort, especially in the face of uninteresting or challenging tasks. The measurement tools used in this study might not have fully captured the nuances of this construct. For instance, the items in the scale may have focused too much on the quantity of effort rather than the quality or the effectiveness of effort allocation. Second, effort regulation is closely intertwined with other factors such as motivation and self-efficacy. In the context of this study, it is possible that the influence of effort regulation on academic success was confounded by these other variables. Future research could consider using more comprehensive and refined measurement instruments that take into account the multi-dimensional nature of effort regulation and control for potential confounding factors. The third reason for the non-confirmation of the relationship between effort regulation and academic success could be related to the sample characteristics. The study sample may have consisted of students with relatively homogeneous levels of effort regulation. If most students in the sample already had a high level of effort regulation, it would be difficult to detect a significant positive relationship with academic success. In such a case, the variance in effort regulation may have been too low to observe its impact on academic success. Future research could expand the sample size and include a more diverse group of students from different educational backgrounds, cultures, and academic levels to increase the variance in effort regulation and potentially uncover its true relationship with academic success.

Regarding the lack of a positive association between management and academic success, one potential factor could be the context of the study which may also play a role in the unexpected results. The study was likely conducted within a specific educational context, which may have unique characteristics that influenced the relationships between the variables. Moreover, the cultural norms and values regarding effort and learning in the study context may have affected how students perceive and regulate their effort, thus altering the expected relationship with academic success. Further research should consider conducting cross-cultural and cross-contextual studies to explore whether the relationships hold in different settings.

Structural equation modeling analysis of the direct effects revealed that metacognitive strategies are relevant to academic procrastination significantly and negatively (Hypothesis 4 was confirmed), and effort regulation was relevant to academic procrastination negatively (Hypothesis 5 was confirmed). There is also a marked negative relationship between time management and academic procrastination (Hypothesis 6 was confirmed). This was in line with the result that academic procrastination affects metacognitive self-regulation negatively (Ziegler and Opdenakker, 2018). The results are also in accord with a previous study that investigated Interactive ambulatory assessment as an approach to encourage self-regulation strategies for combating academic procrastination in the daily learning routine of students, which revealed academic procrastination has a significantly negative relationship with effectively used study time (Loeffler et al., 2019b). This research further disclosed that academic procrastination is directly and negatively related to academic success (Hypothesis 7 was confirmed). This was close to the previous research that academic procrastination was negatively correlated to students’ GPA, and there is a negative relationship between academic procrastination and academic success (Üztemur, 2020).

The present study also studied the indirect effects of metacognitive strategies, effort regulation, and time management on academic success through the mediation of academic procrastination. The results indicated that academic procrastination does not mediate the effects of metacognitive strategies on students’ academic success (Hypothesis 8 was not confirmed). Effort regulation had a considerably positive indirect impact on student’s academic success through the mediation of academic procrastination (Hypothesis 9 was confirmed). Time management had a significantly positive indirect impact on student’s academic success through the mediation of academic procrastination (Hypothesis 10 was confirmed). These results were close to previous research, that is, the motivational regulation strategies have a significant positive and indirect effect on students’ academic performance of via the mediation of academic procrastination.

The contributions of this research to EFL students’ academic success are multi-faceted. First, this research designed a framework for EFL students to better improve their academic success. Second, this framework showed that academic procrastination affects academic success negatively. The reduction of academic procrastination will help increase students’ academic success. Third, this framework found that failure of regulating strategies of metacognitive self-regulation, time management, and effort regulation would have an adverse impact on academic success. Strengthening the time management strategy, the metacognitive strategy, and the effort regulation strategy will promote students’ academic success. Fourth, this framework also showed that better management of regulating strategies would decrease the degree of academic procrastination. Academic procrastination could be a mediation among effort regulation, metacognitive self-regulation, time management, and academic success. When strategies like time management, effort regulation, and metacognitive self-regulation are fostered, then academic procrastination is overcome, so that academic success is promoted.

This study gave new insights into university EFL students’ learning success. All five variables as metacognitive strategies, effort regulation, time management, academic procrastination, and academic success were approached, and academic procrastination as a self-regulatory failure (Katz et al., 2014; Ziegler and Opdenakker, 2018), which has a negative effect with sub-strategies of SRL and students’ academic success. That was to say when the learning strategies like metacognitive strategies, effort regulation, and time management were improved, students’ academic procrastination would reduce. Just because of this, students would complete their learning tasks and assignment better. Therefore, they could achieve better test scores. For the same reason, when the rate of academic procrastination could be reduced means students have used the proper learning strategies, and they can regulate themselves well so that they could achieve success in their learning.

Additionally, EFL learning depends on strategies, the role of the teacher, and student performance (Mohammed, 2018). However, self-regulatory strategies do not develop by all English learners automatically, they can be trained by teachers to stimulate and promote students. In EFL learning, metacognitive strategies can be used by educators or teachers to instruct students to develop proper plans for English Learning (Baron Levi, 2020). It’s also vital for EFL teachers to guide and improve the effort regulation and time management of EFL students.

The contributions of this research to EFL teachers are as follows: first, this framework is skillful for educators or teachers to appropriately utilize these SRL strategies properly. In detail, educators or teachers must apply SRL theories and models to enhance student’s SRL abilities and learning. Self-regulated learning strategies like metacognitive strategies, effort regulation, and time management could be incorporated into teachers’ daily teaching strategies to assist English learners make good learning plans and use the best learning strategies to learn English. Third, teachers or instructors who lead students to use effort regulation, time management, and metacognitive self-regulation strategies effectively can help them overcome academic procrastination in English learning. In conclusion, the more effective the teacher-directed strategy, the less academic procrastination there will be and the better the students’ academic success.

To operationalize the findings, educators can implement targeted, strategy-specific interventions tailored to the identified SRL components: For metacognitive strategies, integrate “think-aloud” modeling into lessons, where teachers verbalize planning (e.g., “before reading this article, I will skim the headings to identify the main topic”), monitoring (e.g., “I notice I’m struggling with this vocabulary; I’ll pause to look up definitions”), and evaluating (e.g., “Does my essay argument align with the prompt?”). Assign reflective journals for students to record how they apply metacognitive strategies (e.g., “I used a summary chart to organize key points in today’s listening task”). For time management, to introduce “time-blocking” templates for EFL tasks (e.g., 20 min for vocabulary review, 30 min for writing practice) and provide visual planners to track process. For procrastination –prone students, use the “5-min rule” (encouraging minimal initial effort to start tasks) paired with incremental goal-setting (e.g., “Complete one paragraph today, expand it tomorrow”). For effort regulation, to design gamified reward systems (e.g., sticker charts for consistent effort) or peer accountability groups where students share weekly goals and celebrate milestones. For challenging tasks (e.g., preparing for a presentation), scaffold effort by breaking them into manageable steps (e.g., research—outline—rehearsal) and offering choice in task formats (e.g., video vs. poster presentations) to boost engagement. These interventions ground SRL theory in daily classroom practice, enabling teachers to systematically build students’ capacity to self-regulate while directly addressing the root causes of academic procrastination in EFL learning.

### 5.2 Research limitations and future recommendations

Despite these promising findings, this research identifies several areas that future studies should address. The first limitation is that this research assessed academic procrastination through a questionnaire and conceptualized procrastination as being relatively stable over situations and time. However, some researchers believe that procrastination is a dynamical, situation-specific, and behavior-related phenomenon. This limitation may affect measurement accuracy and depth, failing to fully represent the complexity of procrastination in students’ real lives, and thus potentially distorting the relationships between procrastination and other variables under investigation. The second limitation is that although the sample size of this study is reasonable for SEM analysis, it would be more accurate if the sample size was larger than 300. Third, there were considerably more female students than male students. This group composition reflects the gender distribution of foreign language majors in Shaanxi Normal Universities, Northwest University, the Xi’an University of Technology, and Baoji University of Arts and Sciences. However, due to potential gender differences in using learning strategies, the findings of this research might not be suitable to samples of students with other gender distributions. Fourth, the current study employed a cross-sectional research design, which failed to account for the sequential nature of variables as required by mediation models. This limitation may hinder the ability to establish clear causal relationships among the variables under investigation.

Future research could consider the following points: First, besides using questionnaires for evaluating academic procrastination, future research should adopt more comprehensive and dynamic approach to measuring procrastination. Second, as Structural Equation Modeling requires at least 200 sample sizes, and the ideal sample size is more than 300, it’s suggested that subsequent studies expand the sample size to obtain better analysis results. Third, considering that the number of female students exceeded that of male students in this study, the sample size of male students can be appropriately increased in future studies. Fourth, mediation models usually imply a sequence of variables, i.e., the independent variables precede the mediation variables, and the mediation variables precede the outcome criteria (Loeffler et al., 2019a; MacKinnon, 2008). Future studies are recommended to assess time management, metacognitive strategies, effort regulation, academic procrastination, and student’s academic success at different time points.

## Data Availability

The original contributions presented in the study are included in the article/supplementary material, further inquiries can be directed to the corresponding author.
